# Mild encephalopathy with a reversible splenial lesion mimicking transient ischemic attack: A case report

**DOI:** 10.1097/MD.0000000000005258

**Published:** 2016-11-04

**Authors:** Kai Dong, Qian Zhang, Jianping Ding, Liankun Ren, Zhen Zhang, Longfei Wu, Wuwei Feng, Haiqing Song

**Affiliations:** a Department of Neurology, Xuanwu Hospital, Capital Medical University, Beijing, China; b Department of Neurology, MUSC Stroke Center, Medical University of South Carolina, Charleston, South Carolina. .

**Keywords:** corpus callosum, reversible splenial lesion syndrome, transient ischemic attack

## Abstract

**Background:**

Reversible splenial lesion syndrome (RESLES) is a newly recognized syndrome, and a reversible progress associated with transiently reduced diffusion lesion in the splenium of the corpus callosum (SCC) is the typical pathological finding. The routine clinical symptoms include mildly altered states of consciousness, delirium, and seizures.

**Methods:**

We presented a 14-year-old patient with signs suggestive of transient ischemic attack (TIA), including triple episodic weakness on the right upper limb, slurred speech, and bucking, lasting several hours in each time 2 days ago. She just had a slight cold 2 weeks ago.

**Results:**

No evidence of abnormality was found in laboratory examinations except an elevated percentage of lymphocyte. Magnetic resonance imaging revealed lesions in splenium of the corpus callosum and frontal-parietal subcortex on both cerebral hemispheres. Cerebrovascular examination was also unremarkable. The patient recovered to baseline within 25 hours. No treatment was given to her in hospital. In addition, the follow-up brain magnetic resonance imaging scan showed reduced lesions. TIA-like symptoms did not occur during a 30-day follow-up period.

**Conclusion:**

This young patient with RESLES type II exhibited TIA-like symptoms, which was not previously reported in literature. This case extends the recognized clinical phenotypes for this disorder.

## 1 Introduction

Reversible splenial lesion syndrome (RESLES) is a newly minted syndrome that was first described by Tada et al in 2004.^[1]^ It is recognized as a new type of acute encephalopathy characterized by a reversible pathological condition associated with transiently reduced diffusion lesion in the splenium of the corpus callosum (SCC).^[2]^ Typically, the clinical symptoms of RESLES include mildly altered states of consciousness, delirium, and seizures after a range of previous viral infections. The common feature is the disappearance of imaging abnormalities and clinical improvement in a few days. The clinical-radiological spectrum of RESLES has been extended since the first report. Combination of reversible lesion in the splenium of corpus callosum and in other brain areas is termed RESLES type II. We here report a patient with RESLES type II, manifesting the transient ischemic attack (TIA)-like symptoms.

## 2 Case report

A 14-year-old girl was admitted to our hospital owing to a history of triple episodic weakness on the right upper limb, slurred speech, and bucking in 2 days, lasting several hours in each time. The previous history is unremarkable except a slight cold 2 weeks ago, with loss of appetite, tiredness, and headache, without fever. The flu-like symptoms resolved completely without any medication.

Only an elevated percentage of lymphocyte (46.8%, normal range: 20%–40%) was found in complete blood cell count. No evidence of abnormality was found in other laboratory examinations, including urine analysis, biochemistries, including erythrocyte sedimentation rate, C-reaction protein, HIV P24 antibody/antigen, Protein S, Protein C, Vitamin B12, and folic acid. The investigation of lumbar puncture was normal, including both counting analysis and biochemistry. TORCH panel just detected negative results for Herpes simplex virus and Cytomegalovirus in cerebrospinal fluid. Cerebrovascular examination was unremarkable, including transcranial color Doppler for intracranial arteries, transcranial Doppler, and magnetic resonance angiogram for carotid artery. Ultrasonic cardiogram ruled out the possibility of patent foramen ovale and other heart diseases.

**Figure 1:**
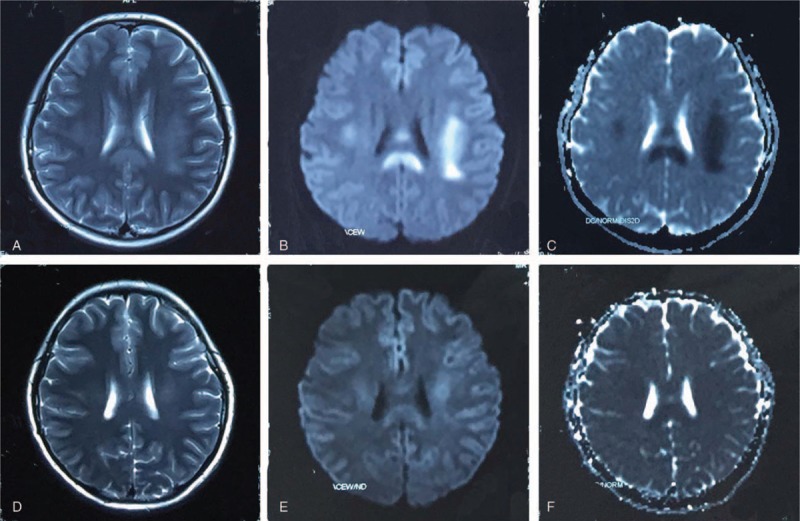
MRI imaging at 24 hours showing increased signals on T2-images in the SCC and frontal-parietal subcortex of both cerebral hemispheres (A). Abnormal signals were also found at DWI (B) and ADC images (C) in the same area. MRI imaging at 10 days later exhibited reduced abnormal signals in T2 (D), DWI (E), and ADC (F) in the same area. ADC = apparent diffusion coefficient, DWI = diffusion-weighted imaging, MRI = magnetic resonance imaging, SCC = splenium of the corpus callosum.

**Figure 2:**
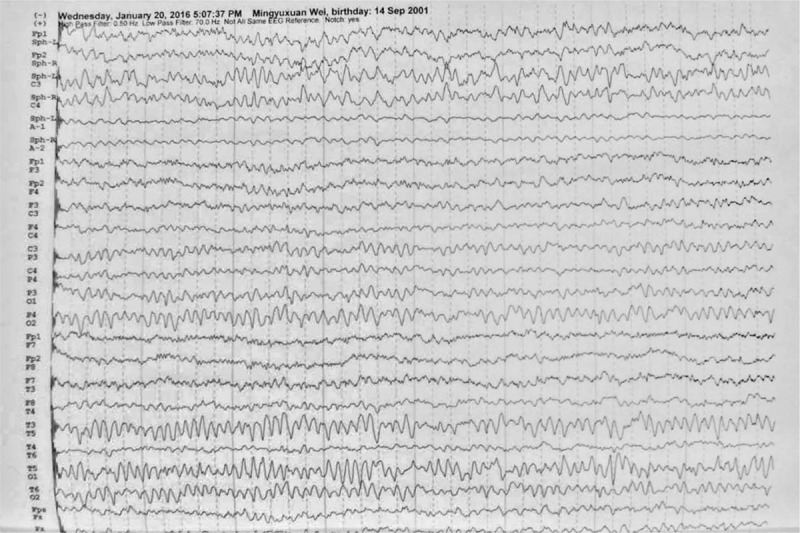
Video electroencephalography examinations: Slow wave in moderate-to-high amplitude occurred following the hyperventilation. A sharp wave occasionally occurred near the left of the sphenoidal electrodes.

The first magnetic resonance imaging (MRI) images (T2) revealed distinct lesions involving white matter in the SCC and frontal-parietal sub-cortex on both cerebral hemispheres approximately 24 hours following the first onset of symptoms (Fig. 1). These lesions showed restricted diffusion with hyperintense signal on diffusion-weighted imaging (DWI) and hypointense signal on apparent diffusion coefficient (ADC) sequence. There is no contrast enhancement. As shown in Figure 2, slow wave in moderate-to-high amplitude (3–5 Hz) was found after the hyperventilation in all video electroencephalographies. A sharp wave was occasionally discovered near the left of the sphenoidal electrodes. This patient recovered to baseline within 25 hours. No treatment was given to her in hospital. A follow-up brain MRI scan was performed 10 days after onset of disease. Reduced lesions with decreased signals were discovered in T2, DWI, and ADC (Fig. 1). Episodic weakness, slurred speech, and bucking did not occur during a 30-day follow-up period.

## 3 Discussion

In this case, we first showed TIA-like symptoms in a child with RESLES type II. In essential, reversible splenial lesion and transient encephalopathy is the hallmark of the diagnosis of the RESLES. Several hypotheses have been proposed for pathogenesis of the specific lesion, such as intramyelinic edema, hyponatremia, axonal damage, and oxidative stress. In this case, the benign neurological signs following a nonspecific cold, in association with an abnormal MRI signal on the splenium and white matter, and rapid clinical recovery met the diagnosis criteria of RESLES type II. Compared to RESLES type I, RESLES type II is a rare condition with a total of 18 patients reported.^[3–4]^ Infective agents are considered to be the inducing factor for RESLES.^[5–7]^ This patient presented with a previous history of cold. The blood test on admission revealed an elevated percentage of lymphocyte, which is supportive to the fact that the patient had viral infection within the past few weeks. It is assumed that the viral infection might be causative for diffuse swelling of the myelin sheath and eventual detachment of each layer. The restriction of water molecule diffusion is believed to cause the lesions seen on the neuroimaging. Lesions resolve as the myelin sheath swelling relieves.

Most importantly, a unique TIA-like symptom in this patient offers new insights into RESLES type II. Clinically, variable symptoms of encephalopathy/encephalitis have been reported for patients with RESLES, including drowsiness, delirium, agitation, or disorientation.^[6–7]^ However, paroxysmal weakness on the right upper limb, slurred speech, and bucking firstly occurred in this patient. In addition, these TIA-like symptoms resolved completely without any medication. The edema on the splenium and white matter are assumed to account for the underlying pathophysiology of this kind of episodic neurological deficit.

RESLES type II patients generally were children and young people, and they usually had a good prognosis based on previous reports.^[3,8]^ No medication was given for this patient, and no neurological symptoms were found when the patient was discharged. The abnormal waves were also observed in this patient as previous RESLES patients.^[9]^ Follow-up MRI scans before discharge revealed a reduced lesion with decreased signal. RESLES patients were given glucocorticoid, antiepileptic drugs, antiviral drugs, intravenous γ-globulin, and/or other therapies not specifically targeted to encephalitis/encephalopathy in a previous report.^[10]^ All patients were reported to have complete resolution of neurological symptoms. There is another report on RESLES patient who has improved under monoclonal antibody therapy. It could be because of the therapy or spontaneously.

## 4 Conclusion

This young case demonstrates that RESLES type II can present TIA-like symptoms, which is not previously reported in literature. This case extends the recognized clinical phenotypes of this disorder. In the future, clinicians can be mindful with TIA-like symptoms in patients with possible RESLES type II.

## 5 Informed consent

The patient's parents provided their written informed consent for the publication of this study.

## Acknowledgements

WF acknowledges grant support from American Heart Association (14SDG1829003) and National Institute of Health (P20GM109040). HS acknowledges grant support from the National Key Research and Development Program of China (2016YFC1300600).
